# Identification of conformational B-cell Epitopes in an antigen from its primary sequence

**DOI:** 10.1186/1745-7580-6-6

**Published:** 2010-10-20

**Authors:** Hifzur Rahman Ansari, Gajendra PS Raghava

**Affiliations:** 1 Bioinformatics Center, Institute of Microbial Technology, Sector 39-A, Chandigarh, India

## Abstract

**Background:**

One of the major challenges in the field of vaccine design is to predict conformational B-cell epitopes in an antigen. In the past, several methods have been developed for predicting conformational B-cell epitopes in an antigen from its tertiary structure. This is the first attempt in this area to predict conformational B-cell epitope in an antigen from its amino acid sequence.

**Results:**

All Support vector machine (SVM) models were trained and tested on 187 non-redundant protein chains consisting of 2261 antibody interacting residues of B-cell epitopes. Models have been developed using binary profile of pattern (BPP) and physiochemical profile of patterns (PPP) and achieved a maximum MCC of 0.22 and 0.17 respectively. In this study, for the first time SVM model has been developed using composition profile of patterns (CPP) and achieved a maximum MCC of 0.73 with accuracy 86.59%. We compare our CPP based model with existing structure based methods and observed that our sequence based model is as good as structure based methods.

**Conclusion:**

This study demonstrates that prediction of conformational B-cell epitope in an antigen is possible from is primary sequence. This study will be very useful in predicting conformational B-cell epitopes in antigens whose tertiary structures are not available. A web server CBTOPE has been developed for predicting B-cell epitope http://www.imtech.res.in/raghava/cbtope/.

## Background

A region or segment of an antigen, recognized by a specific antibody or B-cell is called antigenic region or B-cell epitope. These B-cell epitopes can be categorized into two classes, continuous and discontinuous. A continuous/linear epitope is a segment of consecutive residues in the primary sequence while a discontinuous/conformational epitope is a bunch of residues of an antigen that are far away from each other in the primary sequence but are brought to spatial proximity as a result of polypeptide folding. It is also known that most of the B-cell epitope (~90%) are conformational epitope. Both types of epitopes play an important role in the peptide-based vaccines and disease diagnosis [1,2]. One of the beauties of immune system is that it recognizes the foreign proteins/antigens and generate specific antibody against these antigens. This potential of immune system has been exploited by researchers for designing subunit vaccines [3,4].

In the post genomic era where a large number of pathogens have been completely sequenced, it is crucial to identify B-cell epitope or here after called antibody interacting residues in an antigen for the design of subunit vaccines against these pathogens. In the past several experimental techniques have been developed for mapping antibody interacting residues on an antigen that includes identification of interacting residues from structure of antibody-antigen complexes [5]. One of the popular approaches is overlapping peptide synthesis covering the entire antigen sequence, which identifies mainly sequential epitopes [6]. Mapping of antibody interacting residues has been severely hampered by the costly and time taking process of 3D structure determination. Many tools, covering compilation, visualization and prediction of B and T cell epitopes have been developed [7]. Despite of majority of epitopes being conformational, most of the computational methods and databases centered at the sequential epitopes [8-10]. Linear epitope prediction methods can be categorized into physico-chemical property [11], HMM [12] and ANN based [13]. Many methods are available for antibody interacting residues identification if antigen's or its homolog's tertiary structure is known which in itself is a big limitation. These are based on features like flexibility, solvent accessibility [14,15] and amino acid propensity scales [16]. Earlier researchers created a benchmark dataset from the 3D PDB structures and evaluated several structure-based protein-protein binding site prediction methods which included popular CEP [15] and DiscoTope [16] for predicting immunogenic regions [17]. They opted the definition, that epitope consist of antigen residues in which any atom of the antigen residue is separated from any antibody atom by a distance of ≤ 4Å. They found that the performance of all methods were mediocre and no method could achieve Area under curve (AUC) greater than 0.7. In addition to these a bunch of improved methods have been developed for the prediction of antibody interacting residues if tertiary structure of antigen is known [18-23]. In summary, one needs to determine structure of antigen using crystallography in order to identify antibody interacting residues in antigen. The experimental techniques like crystallography are expensive and time consuming where as functional assays are not reliable enough [5]. Thus there is need to develop alternate technique for predicting antibody interacting residues in a protein.

In this study attempt has been made to predict antibody interacting residues in an antigen from its primary sequence. First we created the patterns of different window lengths from the corresponding amino acid sequences then used the standard binary and physico-chemical profiles of patterns. We have introduced for the first time the concept of composition profile of pattern (CPP) generated through sliding window where the central residue is antibody interacting. These features were used to develop SVM based models to predict antibody interacting residues with high accuracy.

## Methods

### Definition of antibody interacting residues or epitope

There are many levels of antigen-antibody interactions one can obtain from PDB structures. Among these interactions we defined antibody interacting residue as a residue of antigen which is at least one atom separated from an antibody atom by 4Å distance. We borrowed this definition from benchmark paper [17] in order to compare our models with existing methods.

### Datasets

#### Main dataset

We obtained 526 antigenic sequences combined from IEDB database and benchmark dataset [9,17]. Sequence redundancy was removed using program CDHIT [24] at 40% cutoff. Finally we got 187 antigens where no two sequences have more than 40% sequence identity. These antigens have 2261 antibody interacting or 2261 residues are part of conformational B-cell epitope and 107414 amino acid residues were non-antibody interacting from the same antigen sequences.

#### Benchmark Dataset

In addition to main dataset, we also evaluate our models on benchmark dataset [17] which contains 161 protein chains from 144 antigen-antibody complex structures. Finally we got non-redundant set of 52 antigen chains where no two sequences have more than 40% sequence identity. This benchmark dataset of 52 antigens contains 858 antibody interacting and 9366 non-antibody interacting residues.

### Creation of patterns

**Figure 1:**
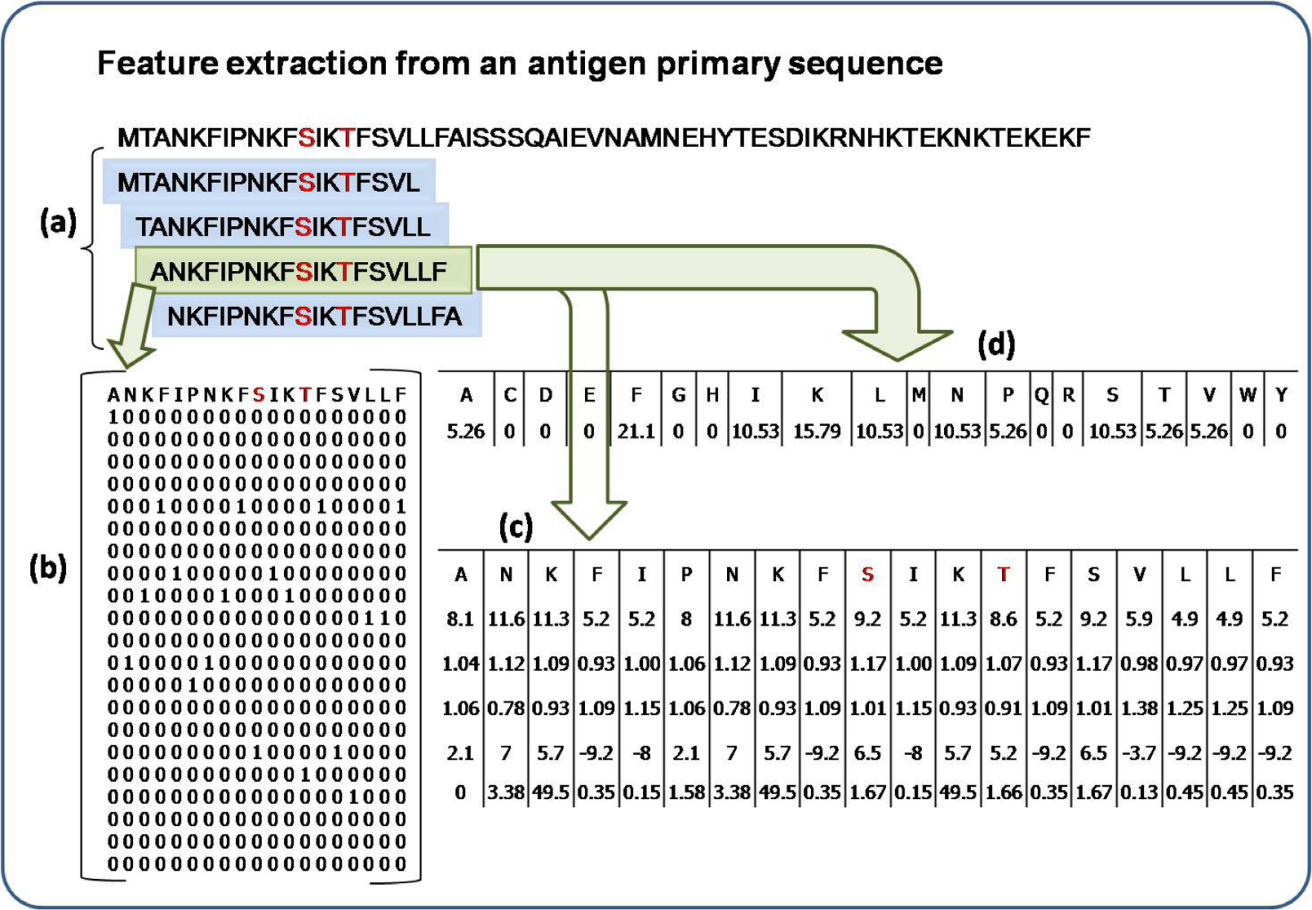
**Feature extraction for a 19 window length pattern**. Antibody interacting residues are marked in red e.g. S/T, Positive pattern shaded in green where S is at the center with 9 neighboring residues on either side, other overlapping negative patterns are shown in blue. a) Creation of 19 window overlapping patterns from amino acid sequence, b) generation of binary profile of pattern (BPP), c) generation of physico-chemical profile (PPP) and d) generation of composition profile of pattern (CPP).

It is known that the function of a residue is not solely determined by itself but influenced by its neighboring residues [25-27]. Thus we generated overlapping patterns of different window sizes from 5 to 21 amino acids for each antigen in the datasets. A pattern is assigned as positive if its central residue interacts with the antibody; else it is assigned as negative (Figure 1). This is the standard procedure used for assigning patterns, which have been used in number of methods like prediction of NAD interacting residues [26], DNA, RNA binding sites in proteins [27], cleavage sites [28] and signal peptides [29]. In order to create a pattern for the terminal residues, we added (L-1)/2 number of dummy residue 'X' on both sides of the protein sequence (L is length of the protein sequence) for e.g. for window size 17 we added 8 'X'.

### Realistic and balance learning

In order to develop prediction method one needs to generate overlapping patterns for each antigen in a dataset; one pattern for each residue. It will produce two types of patterns positive and negative, positive patterns have antibody interacting central residue. These patterns are used to train machine-learning techniques for developing models. In real life only few residues in an antigen are recognized by antibody or B-cell receptor. This means that the number of negative patterns will be much higher than positive patterns in our training dataset; for 2261 positive patterns there were 107414 negative patterns. This creates two problems; i) poor performance of models due to imbalanced set of patterns and ii) training of models is time consuming and CPU intensive. Thus in this study we have used two pattern sets for learning our models; i) realistic set of patterns that includes all negative patterns and ii) balance set of patterns having equal number of positive and negative patterns. In case of balance set, we randomly picked up equal number of negatives from negative pattern set.

### Derivation of features from patterns

#### Binary profile of patterns (BPP)

Each pattern was converted into binary profile, where an amino acid was represented by a vector of dimension 21 (e.g. Ala by 1,0,0,0,0,0,0,0,0,0,0,0,0,0,0,0,0,0,0,0,0). A pattern of window length W was represented by a vector of dimensions 21xW (Additional file 1, Table S1). The binary profile has been used in a number of existing methods [30,31].

#### Physico-chemical profile of patterns (PPP)

As amino acids' physico-chemical properties contribute in the determination of its structure and function, we selected five properties tested by others [32]. These are Grantham polarity [33], Karplus-Schulz flexibility [34], Kolaskar antigencity [35], Parker hydrophobicity [36] and Ponnuswami polarity index [37]. Physico-chemical profile of patterns is similar to the BPP, the only difference lies in the properties of amino acids. Here each amino acid is represented by a vector of 5 i.e. each pattern converted into a vector size of 5xW. For example Ala is represented as [pHydrophobicity, pFlexibility, pPolarity_Grantham, pPolarity_Ponnuswami, pAntigenecity] corresponding to different property values (Additional file 1, Table S2).

#### Composition profile of patterns (CPP)

In the past researchers have exploited amino acid composition of proteins for many biological problems like sub-cellular localization and classification of proteins [38,39]. Instead of calculating composition of antigen sequence, we introduced concept of composition of patterns. The amino acid composition of patterns was calculated using the following equation.

$$comp(i)=\frac{R_{i}}{N}\times 100$$

Where ***comp (i) ***is the percent composition of a residue of type ***i***; ***Ri ***is number of residues of type ***i, ***and ***N ***is the total the number of residues in the pattern.

### Support Vector Machines (SVM)

In the past SVM had been used in a number of biological problems, from classification to functional prediction of proteins [40-42]. In the present study, we have developed a SVM model using a powerful package SVM_light http://svmlight.joachims.org/, for predicting antibody interacting residues in proteins.

### Cross-validation technique

There are many techniques for evaluating the performance of models like leave-one-out or jack-knife test, n-fold cross validation etc [43]. Though jackknife test is the best among cross-validation techniques [44], it is time consuming and CPU intensive technique [40,45]. In order to save time and resources we used widely acceptable 5-fold cross-validation technique. In this technique data is randomly divided into five equal sets of which four sets are used for training and the remaining fifth set for testing. This process is repeated five times in such a way that each set is used once for testing. Final performance is the average of performances achieved on the five sets.

### Performance Measures

The performance of various models developed in this study was computed by using threshold- dependent as well as threshold-independent parameters. In threshold-dependent parameters we used sensitivity (Sen), Specificity (Spe) or percent coverage of non-interacting residues, overall accuracy (Acc) and Matthew's correlation coefficient (MCC) using following equations.

$$Sensitivity=\frac{TP}{TP+FN}\times 100$$

$$Specificity=\frac{TN}{TN+FP}\times 100$$

$$Accuracy=\frac{TP+TN}{TP+TN+FP+FN}\times 100$$

$$MCC=\frac{\left(TP\times TN)-(FP\times FN\right)}{\sqrt{\left[(TP+FN)(TN+FP)(TP+FP)(TN+FN)\right]}}$$

[TP = true positive; FN = false negative; TN = true negative; FP = false positive]

We created ROC (receiver operating curve) for all of the models in order to evaluate performance of models using threshold independent parameters. ROC plots with Area under curve (AUC) were created using SPSS statistical package.

## Results

### Analysis of antibody interacting residues

**Figure 2:**
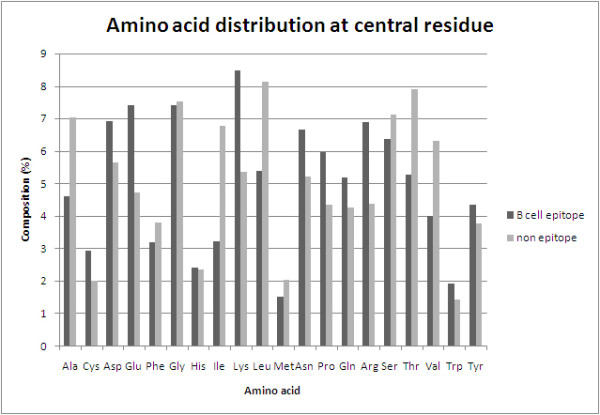
**Comparison of amino acid composition of antibody interacting residues (B-cell epitope) and non-interacting residues (non-epitope)**.

In order to understand whether certain types of amino acids are preferred in antibody interactions, we compared the composition of antibody interacting and non-interacting residues in antigens. As shown in Figure 2, certain types of residues like Cystein, Aspartate, Glutamate, Lysine, Asparagine, Glutamine, Arginine, Trypophan and Tyrosine are preferred in antibody interactions. Most of these are polar and charged residues. In order to understand the preference of interaction in depth, we created 2 Sample Logos [46] for different properties. It was observed that charged, hydrophilic, surface exposed and flexible residues are more abundant in conformational B-cell epitopes (Additional file 1, Figures S1, S2, S3, S4, and S5).

### SVM Models based on BPP and PPP

First, SVM based models have been developed using binary profile of patterns where pattern is represented by a vector of dimensions Nx21 (N is length of pattern). In order to optimize the performance of SVM models, we developed SVM models using patterns of window length 5 to 21. It was observed that models perform better for window size 13, where we got maximum MCC 0.22 with accuracy of 60.84% (Table1). We selected models with minimum difference between sensitivity and specificity. Varying the kernel parameters could not enhance the performance of models and results were just better than random. Detail performance of BPP based SVM model for window length 13 at different thresholds is shown in Additional file 1, Table S3.

**Table 1:** The performance of BPP based SVM model developed using different window lengths from 5 to 21 residues

Window size	Kernel parameters	Thr*	Sen	Spe	Acc	MCC
**5**	t 2 g 0.01 j 1 c 10	0.1	58.38	58.55	58.47	0.17
**7**	t 2 g 0.01 j 1 c 1	0.1	55.87	59.81	57.84	0.16
**9**	t 2 g 0.01 j 1 c 1	0.1	55.66	58.85	57.26	0.15
**11**	t 2 g 0.001 j 1 c 10	0	61.55	56.99	59.27	0.19
**13**	t 2 g 0.1 j 1 c 1	0	62.58	59.09	60.84	0.22
**15**	t 2 g 0.1 j 1 c 10	0	59.93	57.63	58.78	0.18
**17**	t 2 g 0.001 j 1 c 10	0	58.37	57.18	57.78	0.16
**19**	t 2 g 0.001 j 1 c 10	0.1	52.92	63.78	58.35	0.17
**21**	t 2 g 0.001 j 1 c 10	0	59.69	57.22	58.45	0.17

*(Thr- Threshold, Sen - Sensitivity, Spe - Specificity, Acc - Accuracy, MCC - Matthew's correlation coefficient)

It was observed that amino acids having certain types of physico-chemical properties are preferred in antibody interactions (Additional file 1, Figures S1, S2, S3, S4, and S5). Thus we developed SVM based models using PPP and observed best performance for pattern length of 15 residues. As shown in Table 2, we got maximum MCC 0.17 with accuracy 58.31%. The trend and performance of SVM models based on BPP and PPP is similar. Detail performance of PPP based SVM model for window length 15 at different thresholds is shown in Additional file 1, Table S4. Overall performance of PPP based model is slightly poorer than BPP based model (Additional file 1, Tables S3 and S4). All models were trained and tested on main dataset using balance set of patterns.

**Table 2:** The performance of PPP based SVM model developed different window lengths from 5 to 21 residues

W	Kernel parameters	Thr*	Sen	Spe	Acc	MCC
**5**	t 2 g 0.00001 j 1 c 10	-0.3	53.95	59.62	56.78	0.14
**7**	t 2 g 0.00001 j 1 c 10	0.1	55.82	58.03	56.93	0.14
**9**	t 2 g 0.00001 j 1 c 10	0	54.56	55.84	55.2	0.1
**11**	t 2 g 0.00001 j 1 c 10	0.1	52.3	62.48	57.39	0.15
**13**	t 2 g 0.00001 j 1 c 10	0.1	55.11	60.37	57.74	0.16
**15**	t 2 g 0.00001 j 1 c 10	0	56.57	60.06	58.31	0.17
**17**	t 2 g 0.00001 j 1 c 10	0	60.19	55.77	57.98	0.16
**19**	t 2 g 0.00001 j 1 c 10	0	57.82	54.15	55.98	0.12
**21**	t 1 d 1	0	57.31	58.32	57.81	0.16

### SVM Model using Composition Profile of Patterns (CPP)

**Figure 3:**
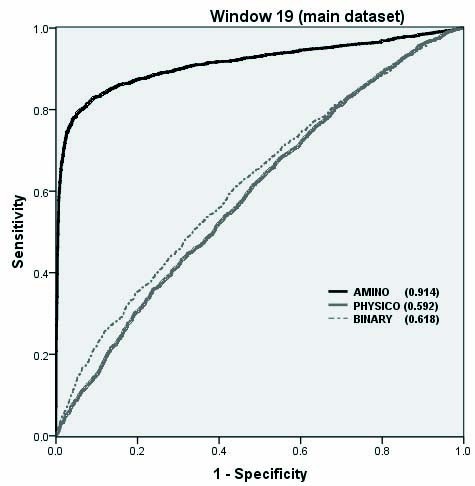
**The performance of SVM models developed using composition, binary and physic-chemical property profile**.

To understand the antibody interacting patterns better, we computed and compared amino acid composition of positive and negative patterns. As shown in Additional file 1, Figure S6, composition profile of positive and negative patterns are different. This means that positive and negative patterns can be discriminated from their amino acid composition. Based on this observation, we developed SVM models for predicting antibody interacting residues in proteins using composition profile of patterns (CPP). The performance of CPP based SVM models have been shown in Table 3. It is surprising that simple composition based model outperforms BPP and CPP based models. We achieved maximum MCC 0.73 with accuracy 86.59% at window length 19. Detail performance of CPP based SVM model for window length 19 is shown in Additional file 1, Table S5. The performance improved significantly for almost all window sizes as compared to binary or physico-chemical properties. As shown in Figure 3, we achieved area under curve (AUC) 0.90 which is significantly better than AUC achieved using BPP and PPP based models. All models were developed from main dataset using balance set of patterns and evaluated using five-fold cross-validation technique.

**Table 3:** The performance SVM models developed using composition profile of patterns at different window lengths

Window size	Kernel parameters	Thr*	Sen	Spe	Acc	MCC
**5**	t 2 g 0.001 j 1 c 1	0	61.75	58.11	59.93	0.2
**7**	t 2 g 0.001 j 1 c 10	0	68.35	62.2	65.27	0.31
**9**	t 2 g 0.001 j 1 c 10	0	73.45	67.21	70.33	0.41
**11**	t 2 g 0.01 j 1 c 1	-0.1	82.08	77.26	79.67	0.59
**13**	t 2 g 0.01 j 1 c 10	-0.1	82.57	84.17	83.37	0.67
**15**	t 2 g 0.01 j 1 c 1	-0.1	79.96	90.31	85.14	0.71
**17**	t 2 g 0.01 j 1 c 1	-0.1	80.69	90.1	85.4	0.71
**19**	**t 2 g 0.01 j 1 c 1**	**-0.1**	**83.13**	**90.06**	**86.59**	**0.73**
**21**	t 2 g 0.01 j 1 c 1	-0.1	83.62	88.96	86.29	0.73

### Comparison with existing methods

**Figure 4:**
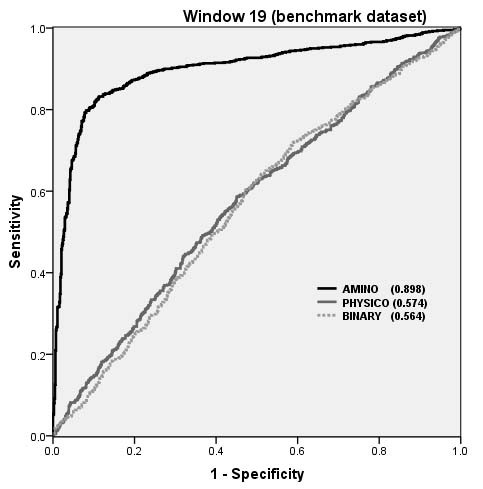
**The performance of SVM models on Benchmark dataset as shown by ROC plot**.

In order to validate our observations, we developed and evaluated our models on benchmark dataset; a dataset used in the past to benchmark earlier methods. All window size patterns were made unique and divided into realistic and balance set of patterns. Realistic set of patterns represents the real-life situation where non interacting residues are much higher than interacting residues. We trained and tested our models on benchmark dataset using balance set of patterns and achieved MCC 0.13 and 0.72 for BPP and CPP respectively (Table 4). These results demonstrates that CPP based models are also effective on benchmark dataset. In order to make evaluation more realistic, we also trained and tested our models using realistic set of patterns based on BPP and achieved MCC 0.06 and 0.44 for BPP and CPP respectively. MCC decreases when we used realistic set of patterns instead of balance set of patterns but accuracy was nearly the same in both cases. In order to compare performance of our model with existing methods we also measured performance in term of AUC. Figure 4 shows the ROC plot of our models on benchmark dataset, we achieved AUC 0.56, 0.57 0.89 for models based on BPP, PPP and CPP respectively. These results demonstrate that CPP based models are more accurate than other models. AUC was more than 0.85 for both set of patterns, realistic and balance (Figure 4). We compared performance of our model with existing methods (Table 5) and observed that our model is as good as any other method. This means our model may complement existing methods and can be used when structure of the antigen is not available.

**Table 4:** The performance of BPP and CPP based SVM model on Benchmark dataset, developed using balance and realistic set of patterns.

Type of Pattern set	Model	SVM parameters	Thr*	Sen	Spe	Acc	MCC
**Realistic**	BPP	t 2 g 0.001 j 10 c 10	-0.2	50.49	60.28	59.49	0.06
	CPP	t 2 g 0.001 j 10 c 10	-0.3	80.41	84.64	84.30	0.44

**Balance**	BPP	t 2 g 0.01 j 1 c 10	0.1	61.31	51.22	56.27	0.13
	CPP	t 2 g 0.01 j 1 c 10	0	82.36	89.42	85.89	0.72

Models were developed using window size 19.

**Table 5:** Overall performance of structure based and CBTOPE algorithms on benchmark dataset

Evaluation parameter	ProMate	PSI-PRED best patch	Patch Dock best model	ClusPro (DOT) best model	CEP	DiscoTope (-7.7)	CBTOPE* (This Study)
Sen*	0.09	0.33	0.43	0.45	0.31	0.42	***0.80***
**1-Spe**	0.08	0.14	0.11	0.07	0.22	0.21	*0.15*
**PPV**	0.10	0.19	0.26	0.39	0.11	0.16	*0.31*
**Acc**	0.84	0.82	0.85	0.89	0.74	0.75	*0.84*
**AUC**	0.51	0.60	0.66	0.69	0.54	0.60	***0.89***

*(Thr- Threshold, Sen - Sensitivity, Spe - Specificity, Acc - Accuracy, PPV - positive predictive value)

### Implementation

A user-friendly web server 'CBTOPE' was developed for the prediction of antibody interacting residues or B-cell conformational epitopes. The server is developed using CGI-Perl script, HTML and installed on a Sun Server (420E) under UNIX (Solaris 7) environment. The user may submit the amino acid sequence(s) in 'FASTA' format. The server generates the 19 window patterns of all submitted sequences, calculates amino acid composition and predicts antibody interacting residues. The output is the amino acid sequence mapped with a probability scale ranging from 0 to 9 for each amino acid. 0 indicates the rarest chance of being that residue in a B-cell epitope and 9 as the most probable. We suggest that for high specificity (high confidence) prediction, user should select the higher threshold value but compromising the sensitivity of prediction. However, for maximum prediction of antibody interacting residues user should opt lower threshold. There is always interplay between sensitivity and specificity. The default threshold was set at -0.3 as at this value, sensitivity and specificity was found equal during the development. Web-server is freely available at http://www.imtech.res.in/raghava/cbtope.

## Discussion

It has been a great challenge for the academicians to devise algorithms and methods for the identification and mapping of potential B-cell epitopes from an antigen sequence. Much effort has been put in trying to predict the conformational B-cell epitope. Previous methods predict conformational B-cell epitopes with reasonably high accuracy, the limitation of these methods is that they require tertiary structure of the antigen. Experimental technique like X-ray crystallography used for determining structure of a protein is costly, tedious and time consuming. To the best of author's knowledge there is no method which can predict conformational B-cell epitopes in an antigen in absence of tertiary structure. There is a need to develop methods for predicting conformational B-cell epitopes in an antigen from its primary sequence. This study describes the method CBTOPE developed for predicting conformational epitopes of antibody interacting residues in antigens. In order to compare performance of our models we chose a benchmark dataset, which was used to evaluate the performance of structure based methods. In order to increase the data we included data from IEDB database. We presumed that the antibody interacting residues are the conformational B-cell epitope residues. We used traditional features of binary and physico-chemical profiles of patterns, evaluated by 5-fold cross validation while using SVM as a classifier. Performance was very poor in BPP models due to the fact that for 21xW vector size only W values represent 1, the rest all are 0 so the noise is more in BPP model. PPP model also could not perform well although it was earlier used for linear and structure based conformational B-cell epitope prediction. From the preliminary analysis of the composition and 2 sample logo plots of positive and negative patterns, it was clear that there is significant difference in the composition and surface propensities of certain residues which can be exploited to discriminate the patterns. Finally we used for the first time, in our study simple amino acid composition model of patterns (CPP) with vector size of 20 which was evaluated on two different datasets. The performance improved significantly and it is interesting to note that it can be used for the prediction of conformational B-cell epitopes despite the fact that in CPP model we lost the amino acid order information unlike BPP. This problem may be equated to the sub-cellular localization of proteins wherein it was observed that simple amino acid composition model perform better than other features. But unlike sub-cellular localization we exploited composition of patterns instead of whole protein sequence. It should be noted that despite the prediction of antibody interacting or individual B-cell epitope residues, being a sequence based method and the lack of 3D structural input, CBTOPE cannot assist in determining the number and distance needed to make an epitope segment in the antigen sequence. This information can be obtained by mapping of the predicted residues on the modeled structure. We hope that the present model is unique in its kind and will compliment the available structure based methods used for the prediction of antibody interacting residues or conformational B-cell epitopes.

## Conclusion

We showed that simple antigen sequence can be used for the prediction of conformational B-cell epitopes and no structure or homology is required. We introduced for the first time concept of local amino acid composition of antigen. We showed that our CPP composition based SVM model outperformed other structure methods with better sensitivity and AUC on the same benchmark dataset.

## Competing interests

The authors declare that they have no competing interests.

## Authors' contributions

HRA carried out the data analysis and interpretation, developed computer programs, wrote the manuscript and developed the web-server. GPSR conceived and coordinated the project, guided its conception and design, helped in the interpretation of data, refined the drafted manuscript and gave overall supervision to the project. Both authors read and approved the final manuscript.

## Supplementary Material

Additional file 1 **Additional file for CBTOPE**. Additional file 1 containing BPP and PPP matrix and detailed threshold-wise results of selected windows and kernels. Click here for file

## References

[B1] Gershoni JM Roitburd-Berman A Siman-Tov DD Tarnovitski Freund N Weiss Y Epitope mapping the first step in developing epitope-based vaccines BioDrugs 2007 21 145 156 10.2165/00063030-200721030-00002 17516710 PMC7100438

[B2] Pomes A Relevant B cell epitopes in allergic disease Int Arch Allergy Immunol 2010 152 1 11 10.1159/000260078 19940500 PMC2956005

[B3] Almagro JC Identification of differences in the specificity-determining residues of antibodies that recognize antigens of different size: implications for the rational design of antibody repertoires J Mol Recognit 2004 17 132 143 10.1002/jmr.659 15027033

[B4] MacCallum RM Martin AC Thornton JM Antibody-antigen interactions: contact analysis and binding site topography J Mol Biol 1996 262 732 745 10.1006/jmbi.1996.0548 8876650

[B5] Van Regenmortel MH Structural and functional approaches to the study of protein antigenicity Immunol Today 1989 10 266 272 10.1016/0167-5699(89)90140-0 2478146

[B6] Frank R The SPOT-synthesis technique. Synthetic peptide arrays on membrane supports--principles and applications J Immunol Methods 2002 267 13 26 10.1016/S0022-1759(02)00137-0 12135797

[B7] Xingdong Y Xinglong Y An introduction to epitope prediction methods and software Reviews in Medical Virology 2009 19 77 96 10.1002/rmv.602 19101924

[B8] Saha S Raghava GP Searching and mapping of B-cell epitopes in Bcipep database Methods Mol Biol 2007 409 113 124 full_text 18449995 10.1007/978-1-60327-118-9_7

[B9] Vita R Zarebski L Greenbaum JA Emami H Hoof I Salimi N Damle R Sette A Peters B The immune epitope database 2.0 Nucleic Acids Res 2010 38 D854 862 10.1093/nar/gkp1004 19906713 PMC2808938

[B10] Saha S Raghava GP Prediction methods for B-cell epitopes Methods Mol Biol 2007 409 387 394 full_text 18450017 10.1007/978-1-60327-118-9_29

[B11] Saha S Raghava GP BcePred: Prediction of continuous B-cell epitopes in antigenic sequences using physico-chemical properties ICARIS, LNCS 2004 3239 197 204

[B12] Larsen JE Lund O Nielsen M Improved method for predicting linear B-cell epitopes Immunome Res 2006 2 2 10.1186/1745-7580-2-2 16635264 PMC1479323

[B13] Saha S Raghava GP Prediction of continuous B-cell epitopes in an antigen using recurrent neural network Proteins 2006 65 40 48 10.1002/prot.21078 16894596

[B14] Novotny J Handschumacher M Haber E Bruccoleri RE Carlson WB Fanning DW Smith JA Rose GD Antigenic determinants in proteins coincide with surface regions accessible to large probes (antibody domains) Proc Natl Acad Sci USA 1986 83 226 230 10.1073/pnas.83.2.226 2417241 PMC322830

[B15] Kulkarni-Kale U Bhosle S Kolaskar AS CEP: a conformational epitope prediction server Nucleic Acids Res 2005 33 W168 171 10.1093/nar/gki460 15980448 PMC1160221

[B16] Haste Andersen P Nielsen M Lund O Prediction of residues in discontinuous B-cell epitopes using protein 3D structures Protein Sci 2006 15 2558 2567 10.1110/ps.062405906 17001032 PMC2242418

[B17] Ponomarenko JV Bourne PE Antibody-protein interactions: benchmark datasets and prediction tools evaluation BMC Struct Biol 2007 7 64 10.1186/1472-6807-7-64 17910770 PMC2174481

[B18] Sweredoski MJ Baldi P PEPITO: improved discontinuous B-cell epitope prediction using multiple distance thresholds and half sphere exposure Bioinformatics 2008 24 1459 1460 10.1093/bioinformatics/btn199 18443018

[B19] Moreau V Fleury C Piquer D Nguyen C Novali N Villard S Laune D Granier C Molina F PEPOP: computational design of immunogenic peptides BMC Bioinformatics 2008 9 71 10.1186/1471-2105-9-71 18234071 PMC2262870

[B20] Huang Y Bao Y Guo S Wang Y Zhou C Li Y Pep-3D-Search: a method for B-cell epitope prediction based on mimotope analysis BMC Bioinformatics 2008 9 538 10.1186/1471-2105-9-538 19087303 PMC2639436

[B21] Huang J Gutteridge A Honda W Kanehisa M MIMOX: a web tool for phage display based epitope mapping BMC Bioinformatics 2006 7 451 10.1186/1471-2105-7-451 17038191 PMC1618411

[B22] Bublil EM Freund NT Mayrose I Penn O Roitburd-Berman A Rubinstein ND Pupko T Gershoni JM Stepwise prediction of conformational discontinuous B-cell epitopes using the Mapitope algorithm Proteins 2007 68 294 304 10.1002/prot.21387 17427229

[B23] Ponomarenko J Bui H-H Li W Fusseder N Bourne P Sette A Peters B ElliPro: a new structure-based tool for the prediction of antibody epitopes BMC Bioinformatics 2008 9 514 10.1186/1471-2105-9-514 19055730 PMC2607291

[B24] Li W Godzik A Cd-hit: a fast program for clustering and comparing large sets of protein or nucleotide sequences Bioinformatics 2006 22 1658 1659 10.1093/bioinformatics/btl158 16731699

[B25] Garnier J Gibrat JF Robson B GOR method for predicting protein secondary structure from amino acid sequence Methods Enzymol 1996 266 540 553 full_text 8743705 10.1016/s0076-6879(96)66034-0

[B26] Ansari HR Raghava GP Identification of NAD interacting residues in proteins BMC Bioinformatics 2010 11 160 10.1186/1471-2105-11-160 20353553 PMC2853471

[B27] Kumar M Gromiha MM Raghava GP Prediction of RNA binding sites in a protein using SVM and PSSM profile Proteins 2008 71 189 194 10.1002/prot.21677 17932917

[B28] Bhasin M Raghava GP Pcleavage: an SVM based method for prediction of constitutive proteasome and immunoproteasome cleavage sites in antigenic sequences Nucleic Acids Res 2005 33 W202 207 10.1093/nar/gki587 15988831 PMC1160263

[B29] Chou KC Shen HB Signal-CF: a subsite-coupled and window-fusing approach for predicting signal peptides Biochem Biophys Res Commun 2007 357 633 640 10.1016/j.bbrc.2007.03.162 17434148

[B30] Xiao X Wang P Chou KC GPCR-CA: A cellular automaton image approach for predicting G-protein-coupled receptor functional classes J Comput Chem 2009 30 1414 1423 10.1002/jcc.21163 19037861

[B31] Xiao X Shao S Ding Y Huang Z Chou KC Using cellular automata images and pseudo amino acid composition to predict protein subcellular location Amino Acids 2006 30 49 54 10.1007/s00726-005-0225-6 16044193 PMC7087770

[B32] Rubinstein ND Mayrose I Martz E Pupko T Epitopia: a web-server for predicting B-cell epitopes BMC Bioinformatics 2009 10 287 10.1186/1471-2105-10-287 19751513 PMC2751785

[B33] Grantham R Amino acid difference formula to help explain protein evolution Science 1974 185 862 864 10.1126/science.185.4154.862 4843792

[B34] Karplus PA Schulz GE Prediction of Chain Flexibility in Proteins - A tool for the Selection of Peptide Antigens Naturwissenschafren 1985 72 212 213 10.1007/BF01195768

[B35] Kolaskar AS Tongaonkar PC A semi-empirical method for prediction of antigenic determinants on protein antigens FEBS Lett 1990 276 172 174 10.1016/0014-5793(90)80535-Q 1702393

[B36] Parker JM Guo D Hodges RS New hydrophilicity scale derived from high-performance liquid chromatography peptide retention data: correlation of predicted surface residues with antigenicity and X-ray-derived accessible sites Biochemistry 1986 25 5425 5432 10.1021/bi00367a013 2430611

[B37] Ponnuswamy PK Prabhakaran M Manavalan P Hydrophobic packing and spatial arrangement of amino acid residues in globular proteins Biochim Biophys Acta 1980 623 301 316 7397216 10.1016/0005-2795(80)90258-5

[B38] Kaundal R Raghava GP RSLpred: an integrative system for predicting subcellular localization of rice proteins combining compositional and evolutionary information Proteomics 2009 9 2324 2342 10.1002/pmic.200700597 19402042

[B39] Bhasin M Raghava GP GPCRpred: an SVM-based method for prediction of families and subfamilies of G-protein coupled receptors Nucleic Acids Res 2004 32 W383 389 10.1093/nar/gkh416 15215416 PMC441554

[B40] Chen C Chen L Zou X Cai P Prediction of protein secondary structure content by using the concept of Chou's pseudo amino acid composition and support vector machine Protein Pept Lett 2009 16 27 31 10.2174/092986609787049420 19149669

[B41] Chen J Liu H Yang J Chou KC Prediction of linear B-cell epitopes using amino acid pair antigenicity scale Amino Acids 2007 33 423 428 10.1007/s00726-006-0485-9 17252308

[B42] Yang ZR Biological applications of support vector machines Brief Bioinform 2004 5 328 338 10.1093/bib/5.4.328 15606969

[B43] Chou KC Zhang CT Prediction of protein structural classes Crit Rev Biochem Mol Biol 1995 30 275 349 10.3109/10409239509083488 7587280

[B44] Chou KC Shen HB Cell-PLoc: a package of Web servers for predicting subcellular localization of proteins in various organisms Nat Protoc 2008 3 153 162 10.1038/nprot.2007.494 18274516

[B45] Chou KC Shen HB A new method for predicting the subcellular localization of eukaryotic proteins with both single and multiple sites: Euk-mPLoc 2.0 PLoS ONE 2010 5 e9931 10.1371/journal.pone.0009931 20368981 PMC2848569

[B46] Vacic V Iakoucheva LM Radivojac P Two Sample Logo: a graphical representation of the differences between two sets of sequence alignments Bioinformatics 2006 22 1536 1537 10.1093/bioinformatics/btl151 16632492

