# Supplementary material for: Identification of conformational B-cell Epitopes in an antigen from its primary sequence
Source: Immunome Res. 2010 Oct 20;6:6. doi: 10.1186/1745-7580-6-6 (PMC2974664; doi:10.1186/1745-7580-6-6)
Supplement: Additional file 1 — Additional file for CBTOPE. Additional file 1 containing BPP and PPP matrix and detailed threshold-wise results of selected windows and kernels. [file 1745-7580-6-6-S1.DOC]

**Additional File 1**

**Identification of conformational B-cell Epitopes in an antigen from its primary sequence**

Hifzur Rahman Ansari and Gajendra P. S. Raghava^§^

Bioinformatics Center, Institute of Microbial Technology, Sector 39-A, Chandigarh, India.

^§^Corresponding author

Email addresses:

HRA: hrahman@imtech.res.in

GPSR: raghava@imtech.res.in

**Additional Tables**

**Table S1:** Vectors use for representing each amino acid for creating binary profile of patterns (BPP). Each residue is represented by a vector of dimension 21 (‘X’ denotes dummy residue).

| **Amino acid** | **BPP Profile** | | | | | | | | | | | | | | | | | | | | |
| --- | --- | --- | --- | --- | --- | --- | --- | --- | --- | --- | --- | --- | --- | --- | --- | --- | --- | --- | --- | --- | --- |
| Ala | 1 | 0 | 0 | 0 | 0 | 0 | 0 | 0 | 0 | 0 | 0 | 0 | 0 | 0 | 0 | 0 | 0 | 0 | 0 | 0 | 0 |
| **Cys** | 0 | 1 | 0 | 0 | 0 | 0 | 0 | 0 | 0 | 0 | 0 | 0 | 0 | 0 | 0 | 0 | 0 | 0 | 0 | 0 | 0 |
| **Asp** | 0 | 0 | 1 | 0 | 0 | 0 | 0 | 0 | 0 | 0 | 0 | 0 | 0 | 0 | 0 | 0 | 0 | 0 | 0 | 0 | 0 |
| **Glu** | 0 | 0 | 0 | 1 | 0 | 0 | 0 | 0 | 0 | 0 | 0 | 0 | 0 | 0 | 0 | 0 | 0 | 0 | 0 | 0 | 0 |
| **Phe** | 0 | 0 | 0 | 0 | 1 | 0 | 0 | 0 | 0 | 0 | 0 | 0 | 0 | 0 | 0 | 0 | 0 | 0 | 0 | 0 | 0 |
| **Gly** | 0 | 0 | 0 | 0 | 0 | 1 | 0 | 0 | 0 | 0 | 0 | 0 | 0 | 0 | 0 | 0 | 0 | 0 | 0 | 0 | 0 |
| **His** | 0 | 0 | 0 | 0 | 0 | 0 | 1 | 0 | 0 | 0 | 0 | 0 | 0 | 0 | 0 | 0 | 0 | 0 | 0 | 0 | 0 |
| **Ile** | 0 | 0 | 0 | 0 | 0 | 0 | 0 | 1 | 0 | 0 | 0 | 0 | 0 | 0 | 0 | 0 | 0 | 0 | 0 | 0 | 0 |
| **Lys** | 0 | 0 | 0 | 0 | 0 | 0 | 0 | 0 | 1 | 0 | 0 | 0 | 0 | 0 | 0 | 0 | 0 | 0 | 0 | 0 | 0 |
| **Leu** | 0 | 0 | 0 | 0 | 0 | 0 | 0 | 0 | 0 | 1 | 0 | 0 | 0 | 0 | 0 | 0 | 0 | 0 | 0 | 0 | 0 |
| **Met** | 0 | 0 | 0 | 0 | 0 | 0 | 0 | 0 | 0 | 0 | 1 | 0 | 0 | 0 | 0 | 0 | 0 | 0 | 0 | 0 | 0 |
| **Asn** | 0 | 0 | 0 | 0 | 0 | 0 | 0 | 0 | 0 | 0 | 0 | 1 | 0 | 0 | 0 | 0 | 0 | 0 | 0 | 0 | 0 |
| **Pro** | 0 | 0 | 0 | 0 | 0 | 0 | 0 | 0 | 0 | 0 | 0 | 0 | 1 | 0 | 0 | 0 | 0 | 0 | 0 | 0 | 0 |
| **Gln** | 0 | 0 | 0 | 0 | 0 | 0 | 0 | 0 | 0 | 0 | 0 | 0 | 0 | 1 | 0 | 0 | 0 | 0 | 0 | 0 | 0 |
| **Arg** | 0 | 0 | 0 | 0 | 0 | 0 | 0 | 0 | 0 | 0 | 0 | 0 | 0 | 0 | 1 | 0 | 0 | 0 | 0 | 0 | 0 |
| **Ser** | 0 | 0 | 0 | 0 | 0 | 0 | 0 | 0 | 0 | 0 | 0 | 0 | 0 | 0 | 0 | 1 | 0 | 0 | 0 | 0 | 0 |
| **Thr** | 0 | 0 | 0 | 0 | 0 | 0 | 0 | 0 | 0 | 0 | 0 | 0 | 0 | 0 | 0 | 0 | 1 | 0 | 0 | 0 | 0 |
| **Val** | 0 | 0 | 0 | 0 | 0 | 0 | 0 | 0 | 0 | 0 | 0 | 0 | 0 | 0 | 0 | 0 | 0 | 1 | 0 | 0 | 0 |
| **Trp** | 0 | 0 | 0 | 0 | 0 | 0 | 0 | 0 | 0 | 0 | 0 | 0 | 0 | 0 | 0 | 0 | 0 | 0 | 1 | 0 | 0 |
| **Tyr** | 0 | 0 | 0 | 0 | 0 | 0 | 0 | 0 | 0 | 0 | 0 | 0 | 0 | 0 | 0 | 0 | 0 | 0 | 0 | 1 | 0 |
| **X** | 0 | 0 | 0 | 0 | 0 | 0 | 0 | 0 | 0 | 0 | 0 | 0 | 0 | 0 | 0 | 0 | 0 | 0 | 0 | 0 | 1 |

**Table S2:** Values of physico-chemical property of amino acids for creating physico-chemical profile of patterns (PPP).

| **Amino acid** | **Property** | | | | |
| --- | --- | --- | --- | --- | --- |
|  | **P1** | **P2** | **P3** | **P4** | **P5** |
| Ala | 8.1 | 1.041 | 1.064 | 2.1 | 0 |
| Cys | 5.5 | 0.96 | 1.412 | 1.4 | 1.48 |
| Asp | 13 | 1.033 | 0.866 | 10 | 40.7 |
| Glu | 12.3 | 1.094 | 0.851 | 7.8 | 49.91 |
| Phe | 5.2 | 0.93 | 1.091 | -9.2 | 0.35 |
| Gly | 9 | 1.142 | 0.874 | 5.7 | 0 |
| His | 10.4 | 0.982 | 1.105 | 2.1 | 51.6 |
| Ile | 5.2 | 1.002 | 1.152 | -8 | 0.15 |
| Lys | 11.3 | 1.093 | 0.93 | 5.7 | 49.5 |
| Leu | 4.9 | 0.967 | 1.25 | -9.2 | 0.45 |
| Met | 5.7 | 0.947 | 0.826 | -4.2 | 1.43 |
| Asn | 11.6 | 1.117 | 0.776 | 7 | 3.38 |
| Pro | 8 | 1.055 | 1.064 | 2.1 | 1.58 |
| Gln | 10.5 | 1.165 | 1.015 | 6 | 3.53 |
| Arg | 10.5 | 1.038 | 0.873 | 4.2 | 52 |
| Ser | 9.2 | 1.169 | 1.012 | 6.5 | 1.67 |
| Thr | 8.6 | 1.073 | 0.909 | 5.2 | 1.66 |
| Val | 5.9 | 0.982 | 1.383 | -3.7 | 0.13 |
| Trp | 5.4 | 0.925 | 0.893 | -10 | 2.1 |
| Tyr | 6.2 | 0.961 | 1.161 | -1.9 | 1.61 |

**P1=** **Polarity (Grantham, 1974)**

**P3=** **Flexibility (Karplus-Schulz, 1985)**

**P3=** A**ntigenicity Kolaskar and Tongaonkar (1990)**

**P4=** **Hydrophilicity scale (Parker et al., 1986)**

**P5=** **Polarity (Ponnuswamy et al., 1980)**

**Table S3:** The performance of BPP based SVM model on main dataset, developed using window length 13. Model was trained using following parameters t=2 (Radial) g=0.1 j=1 c=1.

| **Thr*** | **TP** | **FP** | **FN** | **TN** | **Sen** | **Spe** | **Acc** | **MCC** |
| --- | --- | --- | --- | --- | --- | --- | --- | --- |
| -1 | 2243 | 2173 | 18 | 88 | 99.2 | 3.89 | 51.55 | 0.1 |
| -0.9 | 2226 | 2139 | 35 | 122 | 98.45 | 5.4 | 51.92 | 0.11 |
| -0.8 | 2209 | 2094 | 52 | 167 | 97.7 | 7.39 | 52.54 | 0.12 |
| -0.7 | 2179 | 2019 | 82 | 242 | 96.37 | 10.7 | 53.54 | 0.14 |
| -0.6 | 2137 | 1917 | 124 | 344 | 94.52 | 15.21 | 54.87 | 0.16 |
| -0.5 | 2068 | 1802 | 193 | 459 | 91.46 | 20.3 | 55.88 | 0.17 |
| -0.4 | 1983 | 1672 | 278 | 589 | 87.7 | 26.05 | 56.88 | 0.17 |
| -0.3 | 1888 | 1493 | 373 | 768 | 83.5 | 33.97 | 58.74 | 0.2 |
| -0.2 | 1738 | 1308 | 523 | 953 | 76.87 | 42.15 | 59.51 | 0.2 |
| -0.1 | 1575 | 1120 | 686 | 1141 | 69.66 | 50.46 | 60.06 | 0.21 |
| **0** | **1415** | **925** | **846** | **1336** | **62.58** | **59.09** | **60.84** | **0.22** |
| 0.1 | 1237 | 743 | 1024 | 1518 | 54.71 | 67.14 | 60.92 | 0.22 |
| 0.2 | 1025 | 575 | 1236 | 1686 | 45.33 | 74.57 | 59.95 | 0.21 |
| 0.3 | 829 | 446 | 1432 | 1815 | 36.67 | 80.27 | 58.47 | 0.19 |
| 0.4 | 657 | 317 | 1604 | 1944 | 29.06 | 85.98 | 57.52 | 0.18 |
| 0.5 | 499 | 220 | 1762 | 2041 | 22.07 | 90.27 | 56.17 | 0.17 |
| 0.6 | 364 | 150 | 1897 | 2111 | 16.1 | 93.37 | 54.73 | 0.15 |
| 0.7 | 250 | 92 | 2011 | 2169 | 11.06 | 95.93 | 53.49 | 0.13 |
| 0.8 | 168 | 56 | 2093 | 2205 | 7.43 | 97.52 | 52.48 | 0.11 |
| 0.9 | 107 | 35 | 2154 | 2226 | 4.73 | 98.45 | 51.59 | 0.09 |
| 1 | 62 | 19 | 2199 | 2242 | 2.74 | 99.16 | 50.95 | 0.07 |

*(Thr- Threshold, TP- true positive, FP- false positive, TN- true negative, FN- false negative, Sen – Sensitivity, Spe - Specificity, Acc - Accuracy, MCC - Matthew's correlation coefficient)

**Table S4 -** The performance of PPP based SVM model on main dataset, developed using window length 15. Model was trained using following parameters t=2 (Radial) g=0.00001 j=1 c=10.

| **Thr*** | **TP** | **FP** | **FN** | **TN** | **Sen** | **Spe** | **Acc** | **MCC** |
| --- | --- | --- | --- | --- | --- | --- | --- | --- |
| -1 | 2158 | 1992 | 103 | 269 | 95.44 | 11.9 | 53.67 | 0.13 |
| -0.9 | 2117 | 1926 | 144 | 335 | 93.63 | 14.82 | 54.22 | 0.14 |
| -0.8 | 2061 | 1843 | 200 | 418 | 91.15 | 18.49 | 54.82 | 0.14 |
| -0.7 | 2002 | 1765 | 259 | 496 | 88.54 | 21.94 | 55.24 | 0.14 |
| -0.6 | 1926 | 1663 | 335 | 598 | 85.18 | 26.45 | 55.82 | 0.14 |
| -0.5 | 1843 | 1551 | 418 | 710 | 81.51 | 31.4 | 56.46 | 0.15 |
| -0.4 | 1755 | 1445 | 506 | 816 | 77.62 | 36.09 | 56.86 | 0.15 |
| -0.3 | 1643 | 1314 | 618 | 947 | 72.67 | 41.88 | 57.28 | 0.15 |
| -0.2 | 1514 | 1181 | 747 | 1080 | 66.96 | 47.77 | 57.36 | 0.15 |
| -0.1 | 1403 | 1039 | 858 | 1222 | 62.05 | 54.05 | 58.05 | 0.16 |
| **0** | **1279** | **903** | **982** | **1358** | **56.57** | **60.06** | **58.31** | **0.17** |
| 0.1 | 1185 | 806 | 1076 | 1455 | 52.41 | 64.35 | 58.38 | 0.17 |
| 0.2 | 1051 | 681 | 1210 | 1580 | 46.48 | 69.88 | 58.18 | 0.17 |
| 0.3 | 940 | 579 | 1321 | 1682 | 41.57 | 74.39 | 57.98 | 0.17 |
| 0.4 | 823 | 488 | 1438 | 1773 | 36.4 | 78.42 | 57.41 | 0.16 |
| 0.5 | 698 | 404 | 1563 | 1857 | 30.87 | 82.13 | 56.5 | 0.15 |
| 0.6 | 580 | 335 | 1681 | 1926 | 25.65 | 85.18 | 55.42 | 0.13 |
| 0.7 | 498 | 257 | 1763 | 2004 | 22.03 | 88.63 | 55.33 | 0.14 |
| 0.8 | 410 | 196 | 1851 | 2065 | 18.13 | 91.33 | 54.73 | 0.14 |
| 0.9 | 328 | 148 | 1933 | 2113 | 14.51 | 93.45 | 53.98 | 0.13 |
| 1 | 262 | 119 | 1999 | 2142 | 11.59 | 94.74 | 53.16 | 0.11 |

**Table S5 -** The performance of CPP based SVM model on main dataset, developed using window length 19. Model was trained using following parameters t=2 (Radial) g=0.01 j=1 c=1

| **Thr*** | **TP** | **FP** | **FN** | **TN** | **Sen** | **Spe** | **Acc** | **MCC** |
| --- | --- | --- | --- | --- | --- | --- | --- | --- |
| -1 | 2264 | 2262 | 0 | 2 | 100 | 0.09 | 50.04 | 0.02 |
| -0.9 | 2262 | 2241 | 2 | 23 | 99.91 | 1.02 | 50.46 | 0.06 |
| -0.8 | 2255 | 2216 | 9 | 48 | 99.6 | 2.12 | 50.86 | 0.08 |
| -0.7 | 2244 | 2143 | 20 | 121 | 99.12 | 5.34 | 52.23 | 0.13 |
| -0.6 | 2228 | 2026 | 36 | 238 | 98.41 | 10.51 | 54.46 | 0.19 |
| -0.5 | 2194 | 1818 | 70 | 446 | 96.91 | 19.7 | 58.3 | 0.26 |
| -0.4 | 2150 | 1445 | 114 | 819 | 94.96 | 36.17 | 65.57 | 0.38 |
| -0.3 | 2064 | 829 | 200 | 1435 | 91.17 | 63.38 | 77.27 | 0.57 |
| -0.2 | 1965 | 420 | 299 | 1844 | 86.79 | 81.45 | 84.12 | 0.68 |
| **-0.1** | **1882** | **225** | **382** | **2039** | **83.13** | **90.06** | **86.59** | **0.73** |
| 0 | 1795 | 124 | 469 | 2140 | 79.28 | 94.52 | 86.9 | 0.75 |
| 0.1 | 1702 | 70 | 562 | 2194 | 75.18 | 96.91 | 86.04 | 0.74 |
| 0.2 | 1605 | 46 | 659 | 2218 | 70.89 | 97.97 | 84.43 | 0.72 |
| 0.3 | 1494 | 29 | 770 | 2235 | 65.99 | 98.72 | 82.35 | 0.68 |
| 0.4 | 1326 | 17 | 938 | 2247 | 58.57 | 99.25 | 78.91 | 0.63 |
| 0.5 | 1169 | 12 | 1095 | 2252 | 51.63 | 99.47 | 75.55 | 0.58 |
| 0.6 | 987 | 8 | 1277 | 2256 | 43.6 | 99.65 | 71.62 | 0.52 |
| 0.7 | 770 | 6 | 1494 | 2258 | 34.01 | 99.73 | 66.87 | 0.45 |
| 0.8 | 529 | 2 | 1735 | 2262 | 23.37 | 99.91 | 61.64 | 0.36 |
| 0.9 | 318 | 1 | 1946 | 2263 | 14.05 | 99.96 | 57 | 0.27 |
| 1 | 146 | 0 | 2118 | 2264 | 6.45 | 100 | 53.22 | 0.18 |

**Table S6 -** The performance of CPP based SVM model on benchmark Realistic dataset, developed using window length 19. Model was trained using following parameters t=2 (Radial) g=0.001 j=10 c=10

| **Thr*** | **TP** | **FP** | **FN** | **TN** | **Sen** | **Spe** | **Acc** | **MCC** |
| --- | --- | --- | --- | --- | --- | --- | --- | --- |
| -1 | 751 | 3173 | 71 | 6193 | 91.36 | 66.12 | 68.16 | 0.32 |
| -0.9 | 742 | 2828 | 80 | 6538 | 90.27 | 69.81 | 71.46 | 0.34 |
| -0.8 | 727 | 2548 | 95 | 6818 | 88.44 | 72.8 | 74.06 | 0.36 |
| -0.7 | 718 | 2276 | 104 | 7090 | 87.35 | 75.7 | 76.64 | 0.38 |
| -0.6 | 705 | 2016 | 117 | 7350 | 85.77 | 78.48 | 79.06 | 0.4 |
| -0.5 | 694 | 1790 | 128 | 7576 | 84.43 | 80.89 | 81.17 | 0.41 |
| -0.4 | 677 | 1609 | 145 | 7757 | 82.36 | 82.82 | 82.78 | 0.43 |
| **-0.3** | **661** | **1439** | **161** | **7927** | **80.41** | **84.64** | **84.3** | **0.44** |
| -0.2 | 640 | 1271 | 182 | 8095 | 77.86 | 86.43 | 85.74 | 0.45 |
| -0.1 | 619 | 1141 | 203 | 8225 | 75.3 | 87.82 | 86.81 | 0.45 |
| 0 | 600 | 1009 | 222 | 8357 | 72.99 | 89.23 | 87.92 | 0.46 |
| 0.1 | 582 | 885 | 240 | 8481 | 70.8 | 90.55 | 88.96 | 0.48 |
| 0.2 | 554 | 789 | 268 | 8577 | 67.4 | 91.58 | 89.63 | 0.47 |
| 0.3 | 527 | 692 | 295 | 8674 | 64.11 | 92.61 | 90.31 | 0.48 |
| 0.4 | 479 | 588 | 343 | 8778 | 58.27 | 93.72 | 90.86 | 0.46 |
| 0.5 | 442 | 516 | 380 | 8850 | 53.77 | 94.49 | 91.21 | 0.45 |
| 0.6 | 411 | 435 | 411 | 8931 | 50 | 95.36 | 91.7 | 0.45 |
| 0.7 | 383 | 371 | 439 | 8995 | 46.59 | 96.04 | 92.05 | 0.44 |
| 0.8 | 338 | 317 | 484 | 9049 | 41.12 | 96.62 | 92.14 | 0.42 |
| 0.9 | 298 | 269 | 524 | 9097 | 36.25 | 97.13 | 92.22 | 0.4 |
| 1 | 229 | 208 | 593 | 9158 | 27.86 | 97.78 | 92.14 | 0.34 |

**Table S7 -** The performance of CPP based SVM model on benchmark balanced dataset, developed using window length 19. Model was trained using following parameters t=2 (Radial) g=0.01 j=1 c=10

| **Thr*** | **TP** | **FP** | **FN** | **TN** | **Sen** | **Spe** | **Acc** | **MCC** |
| --- | --- | --- | --- | --- | --- | --- | --- | --- |
| -1 | 819 | 817 | 3 | 5 | 99.64 | 0.61 | 50.12 | 0.02 |
| -0.9 | 818 | 806 | 4 | 16 | 99.51 | 1.95 | 50.73 | 0.07 |
| -0.8 | 818 | 787 | 4 | 35 | 99.51 | 4.26 | 51.89 | 0.12 |
| -0.7 | 813 | 750 | 9 | 72 | 98.91 | 8.76 | 53.83 | 0.18 |
| -0.6 | 802 | 700 | 20 | 122 | 97.57 | 14.84 | 56.2 | 0.22 |
| -0.5 | 793 | 637 | 29 | 185 | 96.47 | 22.51 | 59.49 | 0.28 |
| -0.4 | 782 | 543 | 40 | 279 | 95.13 | 33.94 | 64.54 | 0.37 |
| -0.3 | 763 | 421 | 59 | 401 | 92.82 | 48.78 | 70.8 | 0.46 |
| -0.2 | 740 | 231 | 82 | 591 | 90.02 | 71.9 | 80.96 | 0.63 |
| -0.1 | 706 | 148 | 116 | 674 | 85.89 | 82 | 83.94 | 0.68 |
| **0** | **677** | **87** | **145** | **735** | **82.36** | **89.42** | **85.89** | **0.72** |
| 0.1 | 637 | 62 | 185 | 760 | 77.49 | 92.46 | 84.98 | 0.71 |
| 0.2 | 607 | 52 | 215 | 770 | 73.84 | 93.67 | 83.76 | 0.69 |
| 0.3 | 566 | 45 | 256 | 777 | 68.86 | 94.53 | 81.69 | 0.66 |
| 0.4 | 513 | 34 | 309 | 788 | 62.41 | 95.86 | 79.14 | 0.62 |
| 0.5 | 429 | 24 | 393 | 798 | 52.19 | 97.08 | 74.64 | 0.55 |
| 0.6 | 332 | 17 | 490 | 805 | 40.39 | 97.93 | 69.16 | 0.47 |
| 0.7 | 260 | 11 | 562 | 811 | 31.63 | 98.66 | 65.15 | 0.41 |
| 0.8 | 190 | 6 | 632 | 816 | 23.11 | 99.27 | 61.19 | 0.35 |
| 0.9 | 122 | 5 | 700 | 817 | 14.84 | 99.39 | 57.12 | 0.27 |
| 1 | 55 | 2 | 767 | 820 | 6.69 | 99.76 | 53.22 | 0.18 |

## Additional Figures

**Special Note:** Figure S1 to S5 present positive and negative patterns by Two-sample logos created by 2 Sample Logo webserver [1].

**Figure S1:** Logo created using residues’s charge as a parameter. Positively charged residues (**K, R, H**) are in blue color, negatively charged residues (**D, E**) are colored red; all neutral residues are in black.

**
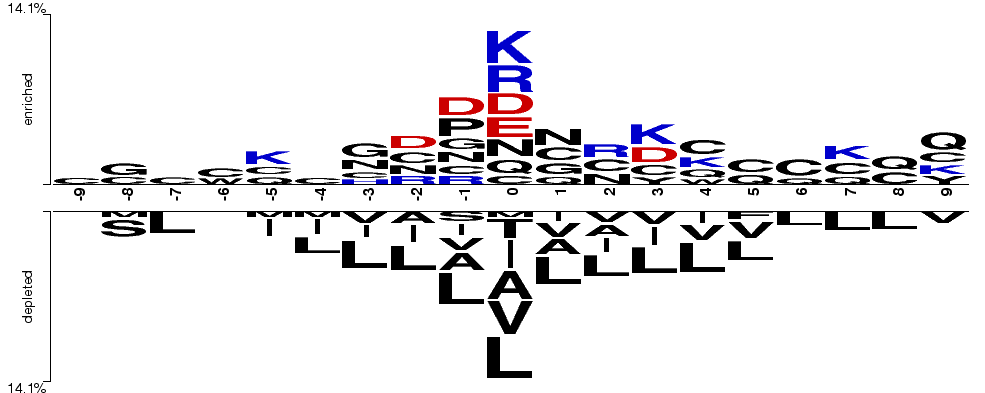
**

**Figure S2:** Logo created using residues’s Hydrphobicity as a parameter. Hydrophobic residues (**A, F, G, I, L, P, V, W, Y**) are cyan colored, while the remaining hydrophilic residues are colored black.

**
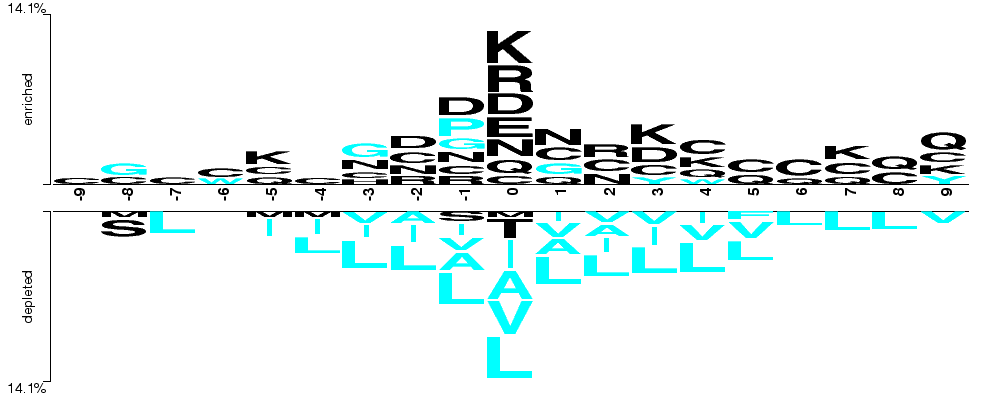
**

**Figure S3**: Logo created using residues’s Surface Exposure as a parameter**.** Surface exposed residues (**D, E, H, K, N, P, Q, R, S, T, Y**) are colored orange, and burried residues (**A, C, F, G, I, L, M, V, W**) are colored black.

**
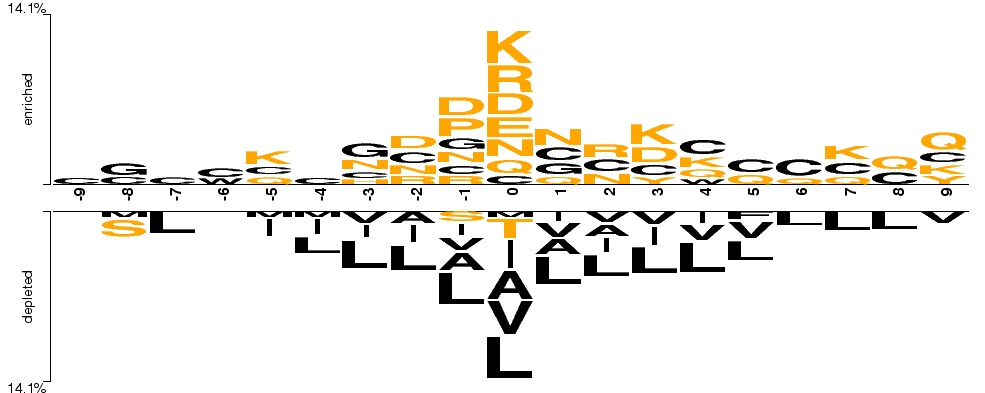
**

**Figure S4:** Logo created using residues’s Flexibility as a parameter**.** High flexibility residues (**D, E, K, N, P, Q, R, S**) are colored red, whereas low flexibility residues (**A, C, F, G, H, I, L, M, T, V, W, Y**) are colored green.


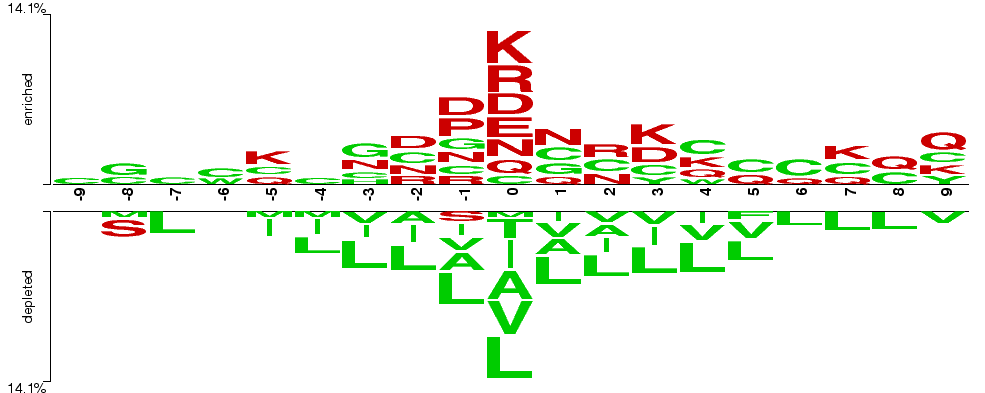


**Figure S5**: Logo created using residues’s Disorderness as a parameter. Disorder-promoting residues (**A, R, S, Q, E, G, K, P**) are colored red, order-promoting residues (**N, C, I, L, F, W, Y, V**) are colored blue, and disorder-order neutral residues (**D, H, M, T**) are colored black.

**
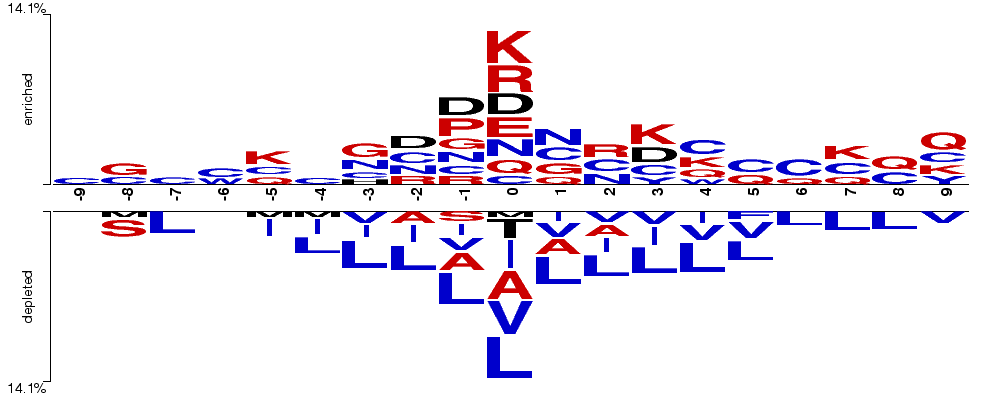
**

**Figure S6:** Comparison of amino acid composition of positive and negative patterns, where central residue of pattern having antibody contacting and non-contacting residue respectively.


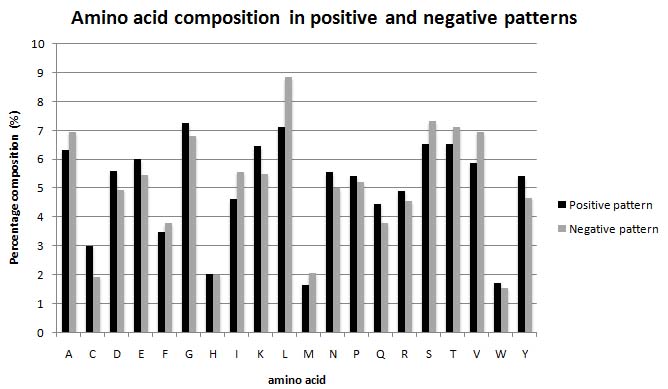


**1. Vacic V, Iakoucheva LM, Radivojac P: Two Sample Logo: a graphical representation of the differences between two sets of sequence alignments*. Bioinformatic*s 2006, 22:1536-1537.**
